# Members of miR-169 family are induced by high salinity and transiently inhibit the NF-YA transcription factor

**DOI:** 10.1186/1471-2199-10-29

**Published:** 2009-04-08

**Authors:** Botao Zhao, Liangfa Ge, Ruqiang Liang, Wei Li, Kangcheng Ruan, Hongxuan Lin, Youxin Jin

**Affiliations:** 1 State Key Laboratory of Molecular Biology, Institute of Biochemistry and Cell Biology, Shanghai Institutes for Biological Sciences, Chinese Academy of Sciences, Shanghai, 200031, PR China; 2 National Key Laboratory of Plant Molecular Genetics, Institute of Plant Physiology and Ecology, Shanghai Institutes for Biological Sciences, Chinese Academy of Sciences, Shanghai 200032, PR China

## Abstract

**Background:**

MicroRNAs (miRNAs) are endogenously expressed small RNAs with a length of about 21 nt. MiRNAs silence their target genes at the post-transcriptional level. In plants, miRNAs play various developmental and physiological roles by cleavaging mRNAs predominantly. Drought and high salinity are the most severe environmental abiotic stresses and cause crop losses all over the world.

**Results:**

In this study, we identified miR-169g and miR-169n (o) as high salinity-responsive miRNAs in rice. MiR-169n and miR169o were in a miRNA cluster with a distance of 3707 base pairs (bp). The high degree of conservation and close phylogenic distance of pre-miR-169n and pre-miR-169o indicated that they were derived from a very recent tandem duplication evolutionary event. The existence of a cis-acting abscisic acid responsive element (ABRE) in the upstream region of miR-169n (o) suggested that miR-169n (o) may be regulated by ABA. In our previous study, we found that miR-169g was induced by the osmotic stress caused by drought via a dehydration-responsive element (DRE). Thus, our data showed that there were both overlapping and distinct responses of the miR-169 family to drought and salt stresses. We also showed that these miR-169 members selectively cleaved one of the NF-YA genes, Os03g29760, which is a CCAAT-box binding transcription factor and participates in transcriptional regulation of large number genes. Finally, we found one or more ath-miR-169 member that was also induced by high salinity.

**Conclusion:**

We identified members of the miR-169 family as salt-induced miRNAs and analyzed their evolution, gene organization, expression, transcriptional regulation motif and target gene. Our data also indicated that the salt-induction of some miR-169 members was a general property in plants.

## Background

MicroRNAs (miRNAs) are a newly identified class of small single-stranded non-coding RNAs that range in length from roughly 18 to 24 nucleotides (nt). Most miRNA genes are thought to exist as independent transcriptional units and are transcribed by RNA polymerase II into long primary transcripts, termed pri-microRNAs [1-4]. Mature miRNA formation is a multi-step process involving many complicated enzymes [5-12]. MiRNAs regulate their target genes via two main mechanisms: target mRNA cleavage and translational repression. In plants, most miRNAs have perfect or near perfect complementarity to their mRNA targets and cleave them [1,13-16]. MiRNAs are involved in plant development, signal transduction, protein degradation, response to environmental stress and pathogen invasion, and they also regulate their own biogenesis [14,16-19]. In the past few years, more and more miRNAs have been identified. However, their functions remain mostly unknown.

Abiotic stresses such as drought, soil salinity, extreme temperatures, chemical toxicity and oxidative stress are serious threats to agriculture and result in environmental deterioration. For plants, drought and high salinity are the main abiotic stresses and are becoming widespread through the world. Many genes and biochemical molecules are involved in the responses to abiotic stress in plants. However there is only a very limited view on miRNAs involved in abiotic stress [20-24]. Rice is a major worldwide crop for food supply, but its yield is frequently affected by abiotic stress. We had analyzed a miRNA profile under drought stress in rice (*Oryza sativa*) and identified two drought-induced miRNAs: miR-169g and miR-393 [22]. MiR-169g was the only member of the miR-169 family induced by drought. Sequence analysis revealed two proximate DREs (Dehydration-Responsive Element) in the upstream region of the *MIR-169g *promoter, suggesting that miR-169g could be regulated directly by the transcriptional factors CBF/DREBs, in response to abiotic stress in plants. These results suggested that miR-169g might play a role under drought stress in rice.

The CCAAT box is one of the most common elements in eukaryotic promoters. It is present in 30% of all eukaryotic genes and is located 80 to 100 base pairs (bp) upstream of the transcription start site (TSS) [25,26]. The NF-Y complex (also known as CBF or HAP) was isolated as a CCAAT-binding protein complex and is an evolutionarily conserved transcription factor that occurs in a wide range of organisms from yeast to humans. It includes three subunits: NF-YA (CBF-B or HAP2), NF-YB (CBF-A or HAP3), and NF-YC (CBF-C or HAP5), all of which are required for DNA binding [25,26]. In *Arabidopsis*, there are 10 genes encoding for the NF-YA subunit, 10 genes for the NF-YB subunit and 9 genes for the NF-YC subunit [27,28]. Further, 10 NF-YA genes, 11 NF-YB genes and 7 NF-YC genes have been identified in rice [29]. All of these plant NF-Y genes contain an evolutionarily conserved domain, which is responsible for DNA binding and protein-protein interactions, and various non-conserved regions in both length and amino acid sequence [25,26].

In this study, we found that not only miR-169g but also miR-169n and miR-169o were induced by high salinity. MiR-169n and miR-169o were located in a miRNA gene cluster and derived from a recent tandem duplication event. There were DRE and ABRE cis-acting elements in the upstream region of miR-169g and miR-169n-o, respectively. We also showed that NF-YA transcription factor, Os03g29760, is a target gene of these salt-inducible miR-169 members and were cleaved rapidly after high salinity treatment. Finally, we found that one or more ath-miR-169 member was also induced by high salinity. This indicated that some members of miR-169 family might be a general property in plants.

## Results

### Response of the miR-169 family to high salinity in rice

**Figure 1:**
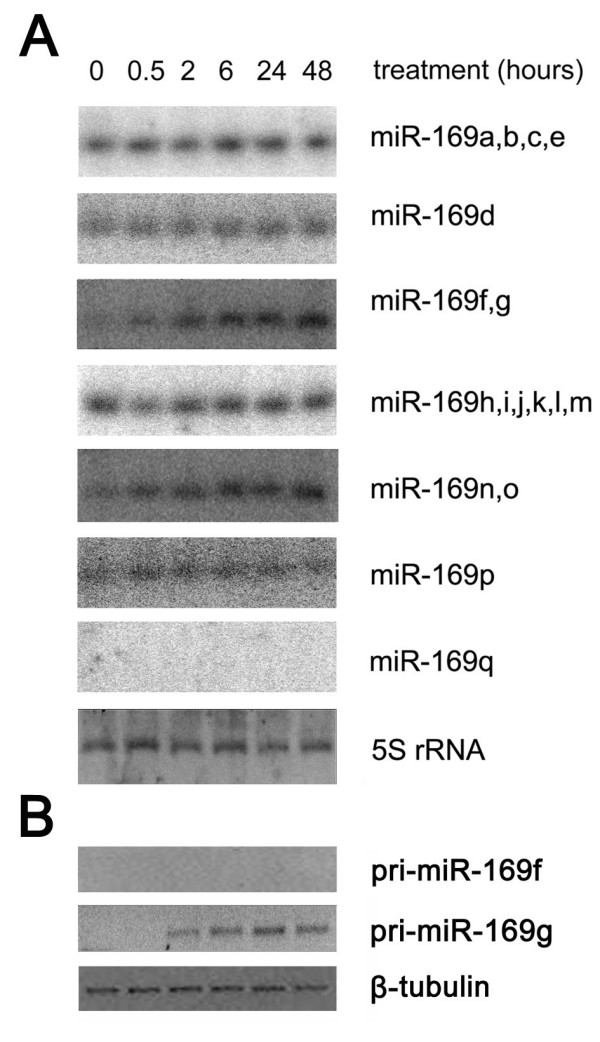
**The miR-169 expression under high salinity**. (A) Rice miR-169 family members were analyzed by northern blot. 5S rRNA stained by ethidium bromide was used as the loading control. (B) RT-PCR analysis of the expression of pri-miR-169f and pri-miR-169g. β-tubulin was used as the interior control.

In our previous study, we identified miR-169g and miR-393 as drought-induced miRNAs in rice [22]. Studies have indicated the existence of significant crosstalk between drought and high-salt stress signaling pathways in plants. Thus, we further tested the response of the miR-169 family to high-salt stress and wondered whether any members of miR-169 family were induced by high salinity. The high salinity environment was mimicked by 150 mM NaCl. The rice seedlings were treated for 0, 0.5, 2, 6, 24 and 48 hours. MiR-169 is a large miRNA family containing 17 known members that represent nine different mature sequences with a little different [22]. Seven probes that had been designed to distinguish these different mature sequences in our previous study were used to screen their responses to high salinity [22]. Two probes (miR-169f, g and miR-169n, o) detected the induced signals (Figure 1A). As in our previous study, the RT-PCR assay indicated that miR-169g, but not miR-169f, was induced by high salinity (Figure 1B). However, the RT-PCR assay failed to distinguish miR-169n and miR-169o because of the high level of conservation in their stem-loop region (see next section). Other members of miR-169 family showed either no induction by high salinity stress or no expression, in keep with our previous report (Figure 1A).

### *MIR-169n *and *MIR-169o *genes were involved in gene duplication evolution

**Figure 2:**
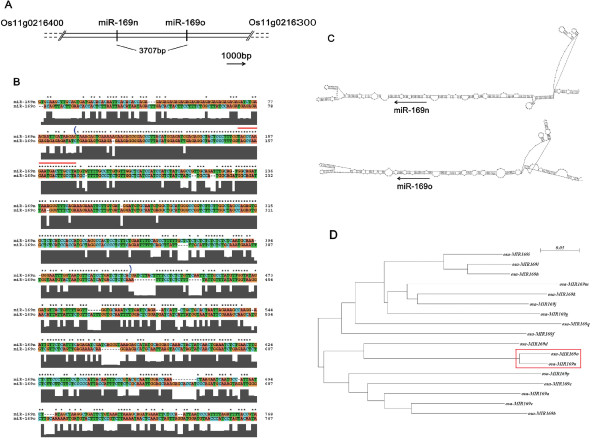
**The conservation of the surround regions of miR-169n and miR-169o**. (A) The cluster was between two annotated genes: Os11g0216400 and Os11g0216300. The distance of miR-169n and miR-169o was 3707 bp. The length scale bar was shown at the bottom right and the orientation of the arrow indicated the direction of transcription. (B) The sequence alignment of the surrounding region of miR-169n and miR-169o. The mature miRNA sequences were labeled by a red line above the sequences. Sequences in the parentheses corresponded to the intact pri-miRNA stem-loops. (C) The intact stem-loops of miR-169n and miR-169o. The mature microRNA sequences were labeled by arrows. The orientation of the arrow indicated the direction of 5' to 3'. (D) The phylogenic tree was shown. miR-169n and miR-169o were indicated by a red frame.

By analyzing the genomic organization and sequence conservation of known miRNA families, several studies had demonstrated that some miRNAs evolved through segmental and tandem duplications in the same manner as protein-coding genes [30-32]. When we analyzed the gene organization of *MIR-169n *and *MIR-169o*, we found the two miRNA genes are clustered in a region of about 10 000 bp between two annotated genes: Os11g0216400 and Os11g0216300. The distance of the two mature miRNAs was 3707 bp (Figure 2A). Furthermore, there was a high degree of conservation in their surrounding regions (Figure 2B). The highest degree of conservation occurred in their stem-loops and slightly less conservation still existed in their downstream regions (Figure 2B). The intact stem-loops of pri-miR-169n and pri-miR169o are very large: more than 300 nt (Figure 2C). Due to the high conservation of their sequences, their predicted secondary structures are also highly similar (Figure 2C). According to the previous studies [30-32], the high conservation in such large and closed regions indicated that *MIR-169n *and *MIR-169o *were the result of a tandem duplication event. Prior to this duplication event, the salt-inducible miRNA was the original microRNA (miR-169no). After duplication, a copy (miR-169n or miR-169o) was generated, and the original and copy formed a miRNA cluster. Moreover, the high level of conservation also suggested that this duplication must have been a recent event. To verify our deduction, we plotted the phylogenic tree of the miR-169 family stem-loops. As expected, the distance between pre-miR-169n and pre-miR-169o was much smaller than any other (Figure 2D). In addition, the branch point of pre-miR-169n and pre-miR-169o emerged much later than others (Figure 2D).

### The cis-acting element analysis of the salt-induced microRNAs

The promoters of drought- and salt-inducible genes contain two major classes of cis-acting elements, ABRE and DRE, both of which are involved in stress-inducible gene expression [33,34]. ABRE and DRE are the major cis-acting elements that function in ABA-dependent and ABA-independent gene expression respectively [33,35].

**Figure 3:**
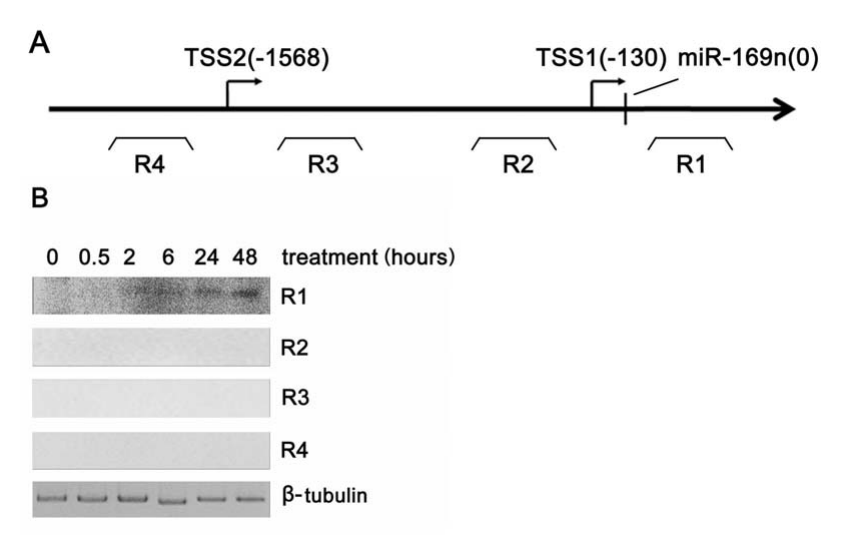
**Mapping the transcription start sites of the miR-169n-o cluster**. (A) The first nucleotide of miR-169n was numbered as zero. R1, R2, R3 and R4 showed the approximate positions of the four regions assayed by RT-PCR. (B) The RT-PCR results from the four regions. β-tubulin was used as the interior control.

To analyze the cis-acting elements of the miR-169n-o cluster, we should first ascertain its potential transcript start site (TSS) and promoter. As previously described, a sequence of approximately 3000 base pairs between the upstream annotated gene and the mature sequence of miR-169n was scanned by TSSP [36]. Two potential promoters were predicted by TSSP, whose TSSs were approximately 130 bp and 1568 bp upstream of the miR-169n (Figure 3A). To test which was the natural promoter, we designed four pairs of primers to detect four different regions around the two promoters (Figure 3A). The results were shown in Figure 3B. Only region 1 (R1) could be amplified successfully from the cDNA. These results indicated that the TSS 130 bp upstream of miR-169n was the genuine one. Then, the 1000 bp upstream region of this promoter was searched for cis-acting elements. There was no DRE in the sequence upstream of the miR-169n-o cluster. However, there was an ABRE at 813 bp upstream of the predicted TSS of the miR-169n-o cluster (Table 1). For the miR-169 family, miR-169g was induced by drought via the DRE, an ABA-independent element. However, miR-169g and miR-169 (o) were induced by high salinity through both DRE and ABRE, where ABRE is an ABA-dependent element (Table 2). These data indicated that the mechanism of the miR-169 response to drought and salt was obviously different.

**Table 1:** ^a ^Cis-acting element analysis of *MIR-169g *and *MIR-169n-o*

**miRNA gene**	**microRNA position^b^**	**DRE (A/GCCGAC)**	**ABRE (ACGTGG/T)**
***MIR-169g***	**85^c^**	**-474, -563**	**Not found**

***MIR-169n(o)***	**131**	**Not found**	**-803**

a. In this table, the numbers denoted the relative positions of the first nucleotide of the mature miRNAs or cis-acting elements to the TSS.

b. The TSS was numbered as +1.

c. Evidence for this TSS was described in (22).

**Table 2:** Comparison of the cis-elements of drought- and salt- induced miRNAs

stress condition	induced miRNA	cis-acting element	pathway
drought	miR-169g	DRE	ABA independent

salt	miR-169g, miR-169n(o)	DRE ABRE	ABA independent, ABA dependent,

### Target genes of the salt-induced miR-169 genes

**Figure 4:**
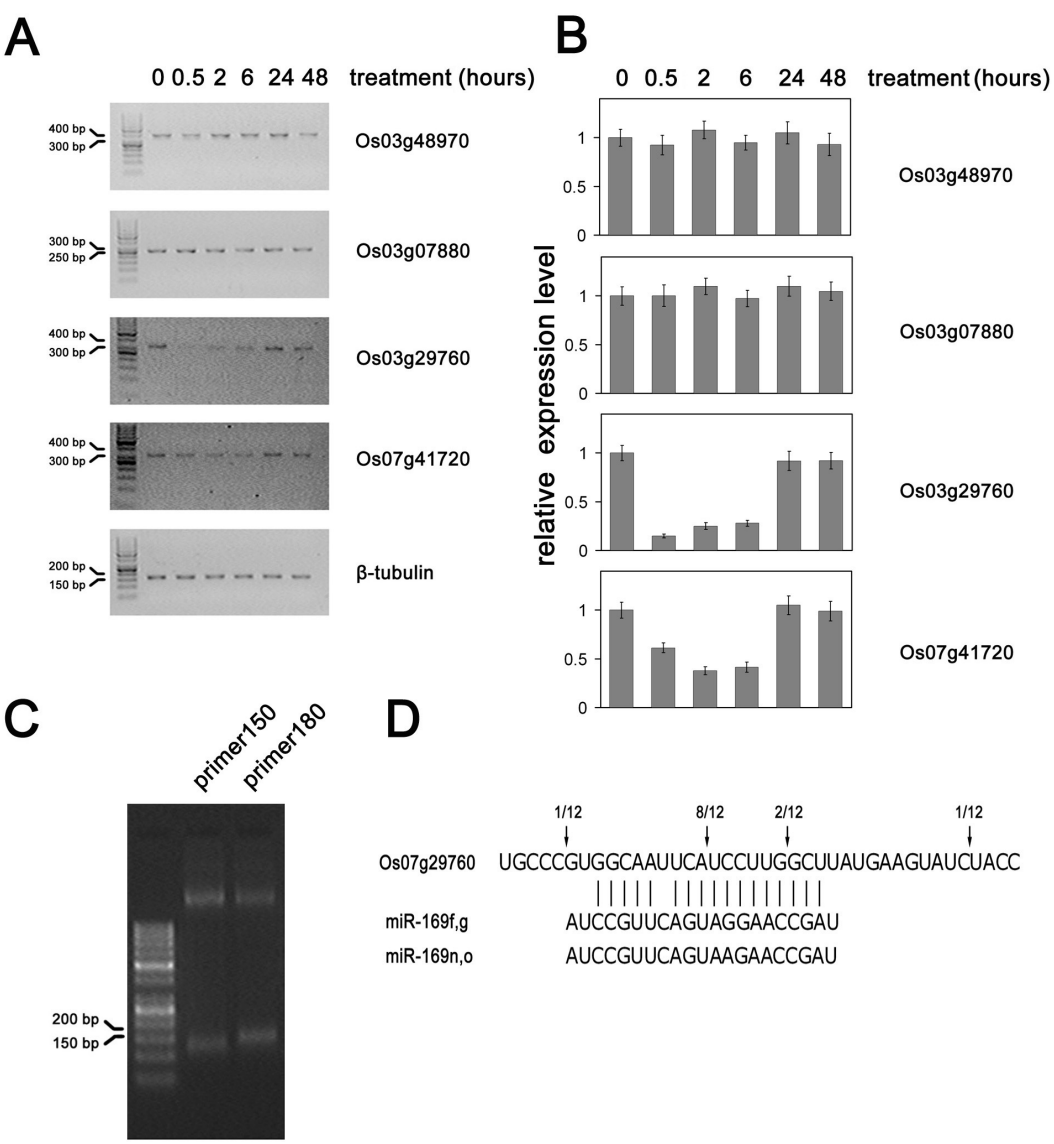
**The Os03g29760 transcript is a target gene of miR-169**. The mRNA levels of the predicted target genes of miR-169 were quantified by RT-PCR (A) and quantitative real-time PCR (B). The mRNA levels of the predicted target genes were quantified by quantitative real-time RT-PCR and normalized to the level of β-tubulin. Error bars represent the standard deviations of three PCR replicates of a single reverse transcription reaction. The normalized mRNA levels in untreated sample were arbitrarily set to 1. (C) The amplified product of the 5' RACE on the Os03g29760 transcript by the primer150 and primer 180. (D) The 5'end of the Os03g29760 determined by 5' RACE.

In *Arabidopsis*, some CCAAT-box binding transcription factors have been reported as target genes of the ath-miR-169 family [4,37]. To identify the target genes of Osa-miR-169, we performed a target gene search by running miRU against the TIGR Rice Genome mRNA database (OSA1 release 5, 01/23/2007) [38]. Consistent with the previous report, several NF-YA genes, a large CCAAT-box binding transcription factor family conserved in all eukaryotes, were predicted to be target genes of miR-169 in rice (see Additional File 1). All of the predicted target sites were located not in the ORF, but in 3'-UTR. Some of them had been recently named by Thirumurugan et al (see Additional File 1) [29]. In their recent genome-wide identification of the NF-Y family, 10 NF-YA genes, 11 NF-YB genes and 7 NF-YC genes were identified in rice [29]. NF-YA is one of the subunits of the NF-Y complex, which bind to the CCAAT-box promoter to regulate the expression of numerous genes. To investigate whether these NY-YA genes were in fact regulated by miR-169, we further quantified the mRNA levels of some predicted genes after high salinity treatment. The primers spanning the target site were designed for RT-PCR. Two amplicons from Os03g29760 and Os07g41720 respectively showed rapid down-regulation after high salinity treatment. The down-regulation of Os03g29760 was more significant and rapid than that of Os07g41720. However, the mRNAs seemed to quickly escape from cleavage and turned to normal level 24 hours after treatment despite the fact that the miRNAs were still induced. Other NF-YA genes had no obvious changes on mRNA levels (Figure 4A). Real-time PCR assay showed similar results (Figure 4B). To further confirm this point, the total RNAs from samples at 0.5 hours, 2 hours and 6 hours were pooled and used for mapping the cleavage site by 5'-RACE assay. As shown in Figure 4C, two expected bands from Os03g29760 were observed by using two Os03g29760 specific primers. The prior sequencing showed that the two bands were the mixtures representing the right cleavage site and some degradation products with the 5' end downstream of the miRNA cleavage site. Those clones with the longest insertions were further sequenced and the results were summarized in figure 4D. However, we failed to amplify any bands from Os07g41720. In all, our data confirmed that Os03g29760 (NF-YA-8 or OsHAP2E), an NF-YA gene, was transiently cleaved by miR-169 after salt treatment in rice.

### The response of the miR-169 family to high salinity in *Arabidopsis*

**Figure 5:**
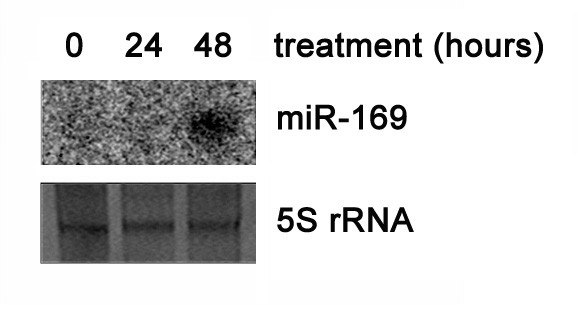
**Ath-miR-169 is induced by salt**. The expression of miR-169 after salt treatment in *Arabidopsis *was determined. 5S rRNA stained by ethidium bromide was used as the loading control.

To determine whether the response of the miR-169 family to high salinity also occurs in other plants, we treated *Arabidopsis *seedling with high salinity. Treatment persisted for 24 hours and 48 hours, as described in the methods section. Because the sequences of the ath-miR-169 family are very similar, only one probe was used for northern blot. Thus, the detected signal was the sum signal from all the miR-169 members. As in rice, the probe to the ath-miR-169 family detected a similar induced signal (Figure 5). This indicated that at least one member of the ath-miR-169 family was induced significantly by high salinity. However, a slight difference between rice and *Arabidopsis *was observed. In rice, the significant induction was observed immediately after 24 hours of treatment, while in *Arabidopsis*, induction was observed until after 48 hours of treatment (Figure 5). This may be attributed to the biological differences among plant species. Of course, the conditions of the plants' culture and treatment could also be one of the causes. These results suggested that the induction of some miR-169 members by abiotic stress was a common phenomenon in plants.

## Discussion

Drought and salinity are becoming particularly widespread in many regions and are the primary cause of crop loss worldwide. Many genes that are induced by these stresses have been identified [39,40]. However, little is known about the relation between the miRNA and these stresses [20-22,24,41]. Our studies have shown that some miR-169 members were induced by these stresses in rice and *Arabidopsis*. Interestingly, it seemed that only a minority of members of the large miR-169 family responded to these stresses. Furthermore, these inducible microRNAs displayed specificity to different stresses.

Plant microRNA families are usually much larger than those of animals. MiR-169 is one of the largest families of miRNAs in plants. To date, the rice miR-169 family contains 17 members in the MicroRNA Registry 10.0 [42,43]. These members have very similar or the same mature sequences and almost the same predicted target genes. However, their expression profiles were very different (Figure 1A). Noticeably, miR-169g and miR-169n (o) exhibited identical responses to high salinity, but different responses to drought. From our studies, we speculated that miR-169 could be a miRNA family that responds to various stress conditions. In our study, the stress condition was more moderate than those of some other groups [21,44,45]. For this reason, we thus could not exclude that other members of miR-169 could be induced by more intense salt stress condition.

At least five signal transduction pathways exist in drought and high salinity stress responses: three are ABA dependent and two are ABA independent [33,34]. In the ABA-dependent pathways, ABRE functions as a major ABA-responsive element. DRE is a major responsive element in the ABA-independent pathway. In this study, high salinity induced both miR-169g and miR-169n (o) which contain DRE and ABRE respectively. In our previous study [22], only drought had induced miR-169g, which contains DREs. PEG treatment only mimics osmotic stress, while salt treatment also brings ionic toxicity except for osmotic stress to plants. From this point, we concluded that miR-169g could have been induced by osmotic stress, while miR-169n and miR-169o were induced by ionic stress. The difference of the cis-acting regulative elements on *MIR-169g *and *MIR-169n (o) *could be part of the molecular base of their diverse responses to stresses.

In addition, we searched the target genes of the miR169 family *in silica*, and genes of NF-YA family were predicted as its target genes. Our subsequent data validated that two of the predicted target genes were down-regulated as the members of the miR-169 were induced by high salinity. NF-YA is one of the subunits of NF-Y complex, which was isolated as a CCAAT-box binding complex and is an evolutionarily conserved transcription factor [25,26]. The CCAAT box is one of the most common elements in eukaryotic promoters and is present in 30% of all eukaryotic genes. A study in *Triticum aestivum *revealed that nine subunits of the NF-Y complex were responsive to drought [46]. *TaNF-YA1 *mRNA levels showed a significant reduction in drought-affected leaves to around one-third of that seen in leaves of the non-stressed control. A similar level of down-regulation in the drought-stressed leaves was seen for four TaNF-YB genes (*TaNF-YB3, 6, 7 and 8*) and three TaNF-YC genes (*TaNF-YC5, 11 and 12*). Only *TaNF-YB2 *was up-regulated under drought condition [46]. That so many NF-Y subunits were down-regulated by drought indicated the expression of many genes controlled by NF-Y complexes were turned off as a result and also that NF-Y complexes should play quite important and comprehensive roles in plant adaptation to drought.

The mRNA levels of Os03g29760 and Os07g41720 declined immediately after high salinity treatment, according to the induction of miR-169 in rice. However, rapid compensation was observed at 24 hours after treatment despite the fact that the miRNAs were still being induced. Our data partially explained the mRNAs reduction of some NF-Y subunits as observed by Stephenson et al [46]. The transient inhibition suggested some mechanisms help the NY-YA gene to escape from cleavage by miR-169 after long-time treatment of high salinity. Given that the NF-Y complex potentially regulates so many genes, we speculated that the roles of miR-169g, miR-169n and miR-169o under salt-stress in rice could be involved in comprehensive physiological processes. Therefore, we thought that these miRNAs are one of the crucial molecules at an upstream node in transcriptional regulatory networks of salt stress signal transduction. In recent years, although both large numbers of up-regulated and down-regulated genes were identified [44,45,47-49], most intention has been focused on genes that were induced in plant under abiotic stresses such as drought and high salinity. The down-regulation of many genes should be also very important for plants to tolerate the environmental stresses, while little is known about that how and why these genes are down-regulated. Our data suggested that the salt-inducible members of the miR-169 family could be pivotal molecules in inhibiting these down-regulated genes under Abiotic stresses.

## Conclusion

In this study, we identified that miR-169g and miR-169n(o) were induced by high salinity. We analyzed the gene organization and conservation of the miR-169n and miR-169o. The high level of conservation suggested that these two miRNAs derived from a tandem duplication event. The promoter searching revealed that there were ABRE elements in the sequence upstream of the miR-169n-o cluster and suggested that miR-169n, o may be regulated by ABA-dependent pathway. We further identified Os03g29760 (NF-YA-8 or OsHAP2E) as a target gene of miR-169 by 5'-RACE. In *Arabidopsis*, at least one member of the ath-miR-169 family was induced significantly by high salinity.

## Methods

### Plant treatments and RNA isolation

The seeds (*Oryza sativa*, var. *japonica*) were stimulated to break dormancy, germinated and then transferred to Yoshida's culture solution under a 13-hour light (26°C)/11-hour dark (22°C) photoperiod. After 12 days in culture, the seedlings were used for experiments. For salt treatment, the seedlings were transferred into fresh culture solution containing 150 mM NaCl. Seedlings were harvested at 0, 0.5, 2, 6, 24 and 48 hours after treatment.

*Arabidopsis *seeds were sterilized and sown on an MS plate containing 0.4% phytage gel and stratified at 4°C for three days, then transferred to 22°C under continuous light for germination and growth. Seven days after germination, the seedings were transferred to a 96-well plate without bottom and grew on MS liquid medium. After 14 days, the MS medium was replaced by MS containing 150 mM NaCl for stress treatment. Seedings were harvested at 0, 24 and 48 hours after treatment.

Samples were then ground to a fine powder and the total RNAs were extracted using the Trizol reagent (Invitrogen).

### RNA gel blot analysis

For mature miRNA analysis, total RNA (10 μg) was loaded per lane and resolved on a denaturing 12% polyacrylamide gel. The gel was separated into two parts. One part containing 5S rRNA was stained by ethidium bromide, and the other containing miRNAs was transferred to a Hybond N+ nylon membrane (Amersham). The membranes were cross-linked with UV at 125 mJ/cm^2^. DNA probes complementary to miRNA sequences were end-labeled with [γ-^32^P] ATP (3000 Ci/mmol) (Amersham) by T4 polynucleotide kinase (MBI). Unincorporated [γ-^32^P] ATP was removed using a sephadex G-25 column. Prehybridization and hybridization were carried out using PerfectHyb Hybridization Solution (TOYOBO) at 37°C. Blots were washed in 2 × SSC/0.1% SDS according to the user manual. The membrane was measured with a PhosphorImager and scanned with GE Storm 860 (Amersham) or FUJIFILM FLA-9000. All probes used were described as previous and listed in Additional File 2. The probe (os-miR-169a, b, c, e) were also used to detect ath-miR-169.

### RT-PCR and quantitative real-time PCR assay

2 μg total RNA and 0.5 μg random hexamers were heated to 70°C for five minutes and then cooled on ice. The remaining reagents (5 × reaction buffer, dNTPs, RNase inhibitor and M-MLV reverse transcriptase (Promega)) were then added and incubated at 37°C for one hour. The RT product was used as a template for PCR using Ex-Taq Hotstart (Takara). The primers used were listed in Additional File 2. To quantify the predicted target genes, these primers were also used for a quantitative real-time PCR assay with SYBR GREEN PCR Master Mix (Applied Biosystems).

### 5'-RACE assay

The total RNA from samples at 0.5, 2 and 6 hours were pooled and used for a 5'-RACE assay. Briefly, total RNA was ligated to a synthesized RNA adaptor (T4 RNA ligase, Fermentas) and then transcribed by gene-specific primers (Superscript II, Invitrogen). The adaptor primer (i.e. the DNA version of the RNA adaptor) and gene-specific primers were used to amplify the ligation product (Ex-Taq Hotstart, Takara). The amplicons were cloned to pMD19-T Vector (Takara). Clones with the expected insertions were sequenced. The sequences of adaptor and primers were listed in Additional File 2.

### Sequence alignment, RNA folding and phylogenic analysis of *Osa-miR-169 *family

The surrounding sequences of miR-169n and miR-169o were extracted and aligned by ClustalX using the default parameter. The conserved sequences (sequences in blue parentheses in Figure 2B) were folded by RNAstructure software. The distances of stem-loops of the *Osa-miR-169 *family stem-loops were calculated with ClustalX and the phylogenic tree was visualized by njplotWin95.

## Authors' contributions

BZ carried out the experiment plan and performed all of experiments except for the plant culture and treatment. LG was responsible for the plant culture and treatment. BZ and LG also wrote the manuscript. Other authors provided helpful suggestions throughout the experiments and reviewed the manuscript. YJ supervised the study and the manuscript writing.

## Supplementary Material

Additional File 1 **Table S1**. Target genes of miR-169g, miR-169n and miR-169o predicted by miRU were validated by RT-PCR. Click here for file

Additional File 2 **Table S2**. Additional File 2. The probes and primers used in this study. Click here for file
