# Supplementary material for: Members of miR-169 family are induced by high salinity and transiently inhibit the NF-YA transcription factor
Source: BMC Mol Biol. 2009 Apr 8;10:29. doi: 10.1186/1471-2199-10-29 (PMC2670843; doi:10.1186/1471-2199-10-29)
Supplement: Additional File 1 — Table S1. Target genes of miR-169g, miR-169n and miR-169o predicted by miRU were validated by RT-PCR. [file 1471-2199-10-29-S1.doc]

**Additional File 1. Target genes of miR-169g, miR-169n and miR-169o predicted by miRU were validated by RT-PCR.**

| ID | Annotation^a^ | Target Site Alignment | Site | Score | Mismatch | Size of RT-PCR fragment | Result of RT-PCR |
| --- | --- | --- | --- | --- | --- | --- | --- |
| Query (3' - 5') | miR-169g | auccguucaguaggaaccgau |  |  |  |  |  |
| Query (3' - 5') | miR-169n and miR-169o | auccguucaguaagaaccgau |  |  |  |  |  |
| Os02g53620| 12002.m10391 | nuclear transcription factor Y subunit A-3 | uaggcaauucauccuuggcuu | 988- 1008 | 1 | 2 | 432bp | - |
| Os03g44540| 12003.m09492 | nuclear transcription factor Y subunit A-10 (HAP2H) | uaggcaaaucauucuuggcuc | 1711- 1731 | 1.5 | 3 | 495bp | - |
| Os12g42400| 12012.m08019 | nuclear transcription factor Y subunit A-2 (OsHAP2F) | uaggcaacucauucuuggcug | 1256- 1276 | 1.5 | 3 | 163bp | - |
| Os03g48970| 12003.m09898 | nuclear transcription factor Y subunit A-1 (OsHAP2D) | caggcaauucauucuuggcuu | 1236- 1256 | 2.5 | 4 | 360bp | + |
| Os03g07880| 12003.m06300 | nuclear transcription factor Y subunit A-3 (OsHAP2C) | auggcaaaucauccuuggcuu | 844- 864 | 3 | 4 | 253bp | + |
| Os03g29760| 12003.m08242 | nuclear transcription factor Y subunit A-8 (OsHAP2E) | guggcaauucauccuuggcuu | 1284- 1304 | 3 | 4 | 310bp | + |
| Os07g41720| 12007.m08408 | nuclear transcription factor Y subunit A-3 (OsHAP2G) | guggcaauucauccuuggcuu | 1169- 1189 | 3 | 4 | 301bp | + |
| Os10g20990| 12010.m05119 | DNA ribosomal protein S12 containing protein | uugguacgucauccuugguug | 1084- 1104 | 3 | 5 | 365bp | - |

a. Gene names in brackets were described in (29).
