# Supplementary material for: Members of miR-169 family are induced by high salinity and transiently inhibit the NF-YA transcription factor
Source: BMC Mol Biol. 2009 Apr 8;10:29. doi: 10.1186/1471-2199-10-29 (PMC2670843; doi:10.1186/1471-2199-10-29)
Supplement: Additional File 2 — Table S2. Additional File 2. The probes and primers used in this study. [file 1471-2199-10-29-S2.doc]

**Additional File 2. The probes and primers used in this study.**

| primer name | sequence |
| --- | --- |
| os-miR-169a, b, c, e probe | TCGGCAAGTCATCCTTGGCTG |
| os-miR-169d probe | CCGGCAATTCATCCTTGGCTA |
| os-miR-169f, g probe | TAGGCAAGTCATCCTTGGCTA |
| os-miR-169h,i, j, k, l, m probe | CAGGCAAGTCATCCTTGGCTA |
| os-miR-169n,o probe | TAGGCAAGTCATTCTTGGCTA |
| os-miR-169p probe | CCGGCAAGTTTGTCCTTGGCTA |
| os-miR-169q probe | CATGGGCAGTCTCCTTGGCTA |
| os-pre-miR-169f forward | CCTGAAGACATGCGTAATGAAGCAGAG |
| os-pre-miR-169f reverse | TGCAGATGAGCAGCGTATAAACACATG |
| os-pre-miR-169g forward | TCATCGATCATCGGTAGGAAGGAAGCT |
| os-pre-miR-169g reverse | TCATGGCACTGCATCATTCAGAAGAG |
| R1 forward | TCTGTTCAATTCTTGCCTATGT |
| R1 reverse | CAGTTGATAGTAGTTTGCTCCTG |
| R2 forward | GTTGCCAACAGTATAGTATTGTTG |
| R2 reverse | GGATGGTCTGTCACATTTCAG |
| R3 forward | TGCTCGCTGTCTCTCATCCT |
| R3 reverse | GGAAAATTGCGAAAGTTTGG |
| R4 forward | CCCATTACTGTCTAACCACAGGT |
| R4 reverse | TCACTACTATAAATGTAAAGGGCGA |
| β-tubulin forward | CCTCCAAGGATTTCAAGTCTGC |
| β-tubulin reverse | TTGTAAGGTTCCACCACGGTA |
| Os02g53620 forward | ACCAAGACCACCACAGACAGC |
| Os02g53620 reverse | AAGCACAGCACTATGAAGCGT |
| Os10g20990 forward | TGGTGGAAACTAGCGAAGTG |
| Os10g20990 reverse | GAAGGGGCGGTGGTAGAC |
| Os03g44540 forward | ACAACAGCATCGACCACCAC |
| Os03g44540 reverse | CAAAGAGCAACCTGACACCA |
| Os03g48970 forward | CTCTTGAGGGTGGATTCTTGC |
| Os03g48970 reverse | AGTTCTGCTCTGGCCTTGTTA |
| Os12g42400 forward | AAGCATGACTAGAGGGGGGA |
| Os12g42400 reverse | AAGTAGGCGATGAGCGGAAC |
| Os03g07880 forward | CTCAACACAAAGCAGCAGCC |
| Os03g07880 reverse | CACAAAACCCACCCATACCC |
| Os03g29760 forward | AGGTGAAAACCTCGGGAT |
| Os03g29760 reverse | TAAGGCAACTTGGTAATGGG |
| Os07g41720 forward | CCACAATGAGCTTCAGAGC |
| Os07g41720 reverse | CATCCACAAACAGAACCAAC |
| RNA adaptor | ACACUGACAUGGACUGAAGGAG |
| DNA adaptor primer | ACACTGACATGGACTGAAGGAG |
| Os03g29760 RACE RT&PCR pimer-150^a^ | TGCTCGACAGTTCGTTCAGTCA |
| Os03g29760 RACE RT&PCR pimer-180 ^a^ | CAGGATTTCTGTACCCACAACAT |
| Os07g41720 RACE RT&PCR pimer-80 ^a^ | GGTCCATTTGAGGATGAAACTACT |
| Os07g41720 RACE RT&PCR pimer-150 ^a^ | AGTCAGTTGATCATTGCAGTGCT |

a. The number suffixes indicated the size of the expected amplicons.
